# Analysis of the *Zonula occludens* Toxin Found in the Genome of the Chilean Non-toxigenic *Vibrio parahaemolyticus* Strain PMC53.7

**DOI:** 10.3389/fcimb.2020.00482

**Published:** 2020-09-24

**Authors:** Diliana Pérez-Reytor, Alequis Pavón, Carmen Lopez-Joven, Sebastián Ramírez-Araya, Carlos Peña-Varas, Nicolás Plaza, Melissa Alegría-Arcos, Gino Corsini, Víctor Jaña, Leonardo Pavez, Talia del Pozo, Roberto Bastías, Carlos J. Blondel, David Ramírez, Katherine García

**Affiliations:** ^1^Facultad de Ciencias de la Salud, Instituto de Ciencias Biomédicas, Universidad Autónoma de Chile, Santiago, Chile; ^2^Facultad de Ciencias Veterinarias, Instituto de Medicina Preventiva Veterinaria, Universidad Austral de Chile, Valdivia, Chile; ^3^Facultad de Ciencias, Centro Interdisciplinario de Neurociencias de Valparaíso, Universidad de Valparaíso, Valparaíso, Chile; ^4^Facultad de Medicina Veterinaria y Agronomía, Universidad de las Américas, Santiago, Chile; ^5^Departamento de Ciencias Químicas y Biológicas, Universidad Bernardo O'Higgins, Santiago, Chile; ^6^Instituto de Ciencias Naturales, Universidad de Las Américas, Santiago, Chile; ^7^Centro Tecnológico de Recursos Vegetales, Escuela de Agronomía, Universidad Mayor, Huechuraba, Chile; ^8^Laboratorio de Microbiología, Instituto de Biología, Pontificia Universidad Católica de Valparaíso, Valparaíso, Chile; ^9^Facultad de Medicina y Facultad de Ciencias de la Vida, Instituto de Ciencias Biomédicas, Universidad Andrés Bello, Santiago, Chile

**Keywords:** *Vibrio parahaemolyticus*, non-toxigenic strains, *Zonula occludens* toxin, Zot, *Vibrio cholerae*, *Campylobacter concisus*, intestinal permeability, Protein structure prediction

## Abstract

*Vibrio parahaemolyticus* non-toxigenic strains are responsible for about 10% of acute gastroenteritis associated with this species, suggesting they harbor unique virulence factors. *Zonula occludens* toxin (Zot), firstly described in *Vibrio cholerae*, is a secreted toxin that increases intestinal permeability. Recently, we identified Zot-encoding genes in the genomes of highly cytotoxic Chilean *V. parahaemolyticus* strains, including the non-toxigenic clinical strain PMC53.7. To gain insights into a possible role of Zot in *V. parahaemolyticus*, we analyzed whether it could be responsible for cytotoxicity. However, we observed a barely positive correlation between Caco-2 cell membrane damage and Zot mRNA expression during PMC53.7 infection and non-cytotoxicity induction in response to purified PMC53.7-Zot. Unusually, we observed a particular actin disturbance on cells infected with PMC53.7. Based on this observation, we decided to compare the sequence of PMC53.7-Zot with Zot of human pathogenic species such as *V. cholerae, Campylobacter concisus, Neisseria meningitidis*, and other *V. parahaemolyticus* strains, using computational tools. The PMC53.7-Zot was compared with other toxins and identified as an endotoxin with conserved motifs in the N-terminus and a variable C-terminal region and without FCIGRL peptide. Notably, the C-terminal diversity among Zots meant that not all of them could be identified as toxins. Structurally, PMC53.7-Zot was modeled as a transmembrane protein. Our results suggested that it has partial 3D structure similarity with *V. cholerae*-Zot. Probably, the PMC53.7-Zot would affect the actin cytoskeletal, but, in the absence of FCIGRL, the mechanisms of actions must be elucidated.

## Introduction

Inshore marine waters around the world are densely populated with *Vibrio parahaemolyticus*, which is the leading cause of seafood-associated bacterial gastroenteritis (Raghunath, 2014; Letchumanan et al., 2017), even though few strains can cause infections in humans and most environmental strains are non-pathogenic (Shinoda, 2011). The most characteristic virulence-associated factors are thermostable direct hemolysin (TDH) and TDH-related hemolysin (TRH), encoded by the *tdh* and *trh* genes, respectively (Nishibuchi et al., 1992; Shinoda, 2011; Zhang and Orth, 2013; Raghunath, 2014), although other virulence factors such as the type III secretion systems of both chromosomes (T3SS1 and T3SS2) and several genomic islands (VPaIs) have been identified (Broberg et al., 2011; Yu et al., 2012; Ceccarelli et al., 2013). Various studies have reported that isolates of non-toxigenic *V. parahaemolyticus*, named like that because of the lack of *tdh, trh*, and T3SS2, can be highly cytotoxic to human gastrointestinal cells (Mahoney et al., 2010; Castillo et al., 2018a), suggesting that other virulence factors must exist (Pérez-Reytor and García, 2018; Wagley et al., 2018). In Chile, the disappearance of the pandemic strain from coasts was associated with a severe diminishing of clinical cases; however, *V. parahaemolyticus* is still considered a significant pathogen associated with food-borne diseases (MINSAL, 2017).

In our recent work, we identified prophages encoding putative *zonula occludens* toxins (Zots) in the genome of highly cytotoxic southern Chilean *V. parahaemolyticus* strains, including the clinical non-toxigenic strain PMC53.7, which does not possess any other known virulence factor in its genome (Castillo et al., 2018a). In *Vibrio cholerae*, Zot is the most important toxin in the absence of the classical cholera toxin (CT), and it is encoded by the CTX prophage (Fasano, 2002; Schmidt et al., 2007; Castillo et al., 2018b). The N-terminal domain of the *V. cholerae*-Zot protein is involved in bacteriophage morphogenesis, while the C-terminal domain is cleaved and secreted into the intestinal lumen (Uzzau et al., 2001; Schmidt et al., 2007; Mahendran et al., 2016). Structure-function analyses indicate that the biologically active fragment of Zot (FCIGRL) can be mapped to amino acids 288–293. The FCIGRL fragment is structurally similar to another motif (SLIGRL) that activates an intracellular signaling pathway by binding to proteinase-activated receptor-2 (PAR-2). This receptor has been implicated in the regulation of paracellular permeability, inducing a transient reduction in the transepithelial resistance and an increase in transepithelial flux along concentration gradients by affecting the tight junction (TJ) (Fasano et al., 1995; Gopalakrishnan et al., 2009; Goldblum et al., 2011; Vanuytsel et al., 2013). Notably, it has been shown that IEC6 cell monolayers treated with *V. cholerae*-Zot in its supernatant displayed a redistribution of actin cytoskeleton, decreasing G-actin while increasing F-actin and the disturbance of paracellular permeability (Fasano et al., 1995). Also, it was reported that toxigenic *Campylobacter concisus* strains producing Zot have the potential to initiate inflammatory bowel disease or could be aggravators of Crohn's disease (Kaakoush et al., 2014; Zhang et al., 2014). This Zot protein causes sustained intestinal barrier damage, induces the release of proinflammatory cytokines, and increases the response of macrophages to other microorganisms (Mahendran et al., 2016). Although there are no reports assigning a role of Zot in the *V. parahaemolyticus* virulence, 77.9% of the clinical isolates of *V. parahaemolyticus* possess *Zot*-encoding prophages, including the f237 of the pandemic RIMD2210633 reference strain (VpKX) (Castillo et al., 2018b). These prophages belong to the Inoviridae family and play an important role in the evolution and pathogenesis of multiple bacterial species (Castillo et al., 2018b).

In this work, we proposed that PMC53.7-Zot is an endotoxin with conserved motifs in the N-terminal end, probably anchored to the membrane and with a structure similar to Zot of other human pathogenic strains. It would be associated with the actin cytoskeletal disturbances observed in PMC53.7-infected Caco-2 cells and with the purified PMC53.7-Zot. However, the mechanisms of action of this toxin and its effects on the intestinal barrier will be the subject of future research.

## Materials and Methods

### Bacterial Strains and Cell Culture

*V. parahaemolyticus* clinical strain PMC53.7 (Harth et al., 2009) and VpKX (Fuenzalida et al., 2006) strains were cultured overnight at 37°C with shaking in Luria-Bertani (LB) broth containing 3% NaCl. The PMC53.7 strain was used to infect Caco-2 cells, as a mammalian intestinal epithelium cell model. The Caco-2 cells are human colonic adenocarcinoma cells that physiologically mimic the mature small intestine villous epithelium (Hidalgo et al., 1989). The cells were grown in Eagle's minimal essential medium (MEM; Sigma-Aldrich, St. Louis, MO, USA) supplemented with 10% fetal bovine serum (FBS; Gibco®, Grand Island, NY, USA) plus 1% antibiotic (Pen-strep; Gibco®, Grand Island, NY, USA). They were maintained in 75 cm^2^ flasks at 37°C under a 5% CO_2_ atmosphere in a humidified incubator until semiconfluence.

### Fluorescent Staining of Cells Infected With Bacteria

The Caco-2 cells were grown on glass slides until ~70% of confluence for staining analysis. Cells were incubated with *V. parahaemolyticus* PMC53.7 and VpKX bacterial culture at a multiplicity of infection (MOI) = 10 or H_2_O_2_ 1 mM. After 3 h (post infection), the slides were washed three times with phosphate-buffered saline (PBS), fixed in paraformaldehyde (PFA) 4% with PBS for 20 min at room temperature, permeabilized with Triton X-100 0.1%, and blocked with 1.0% (w/v) BSA. The F-actin was counterstained using rhodamine phalloidin (Cytoskeleton Inc., Denver, CO, USA) at a dilution of 1:200 in PBS, and nuclei were counterstained with Hoechst stain (H6024; Sigma-Aldrich, St. Louis, MO, USA) solution at 1:5,000 dilution in PBS. The slides were carefully mounted on coverslips and analyzed with an epifluorescence microscope (Leica LX6000, Germany).

### Cytotoxicity Assay

The cellular membrane damage was measured by the release of lactate dehydrogenase (LDH) into the supernatants, using the CytoTox 96 Non-Radioactive Cytotoxicity Assay kit (Promega, Madison, WI, USA) according to the manufacturer's guidelines. The percentage of cytotoxicity was calculated with the equation described by Tanabe et al. (2015). All the experiments were done in triplicate and repeated three times.

### Infection Assay and Zot mRNA Expression

The Caco-2 cells were seeded in a six-well plate (5 × 10^6^ cells per well) and incubated in MEM (Sigma-Aldrich, St. Louis, MO, USA) supplemented with 10% FBS until ~80–90% of confluence. The growth media was removed from monolayers, and cells were washed three times with PBS. A culture in exponential phase (OD_600_ = 0.6) of *V. parahaemolyticus* PMC53.7 strain was centrifuged, and subsequently, a bacterial suspension was prepared in MEM (Sigma-Aldrich, St. Louis, MO, USA), without phenol red and antibiotics, at an MOI = 10, previously standardized. At the onset of infection, cells were centrifuged at 250 g for 4 min to synchronize cell–cell and incubated for 4 h at 37°C and 5% CO_2_. The supernatants and the cells were collected post infection, at 1, 2, 3, and 4 h. The cellular membrane damage was measured by the release of LDH, as described above. All the experiments were done in triplicate and repeated three times. Total RNA from supernatants and cells were isolated with the E.Z.N.A. total RNA kit (Omega Bio-tek, GA, USA) according to the manufacturer's instructions and quantified using an Infinite M200 PRO spectrophotometer (Tecan Austria GmbH). The complementary DNA (cDNA) was synthesized through random hexamer-primed reactions using ImProm-II Reverse Transcriptase (Promega, Madison, WI, USA), according to the manufacturer's instructions, except that we treated the RNA with DNase for twice the time recommended by the kit. Then, the PMC53.7-*Zot* product was analyzed in a Roche LC480 Real-Time PCR system (Roche Diagnostics, Nederland, BV) using Brilliant SYBR Green II single-step quantitative RT-PCR (qRT-PCR) Master Mix (Stratagene–Agilent Technologies, La Jolla, CA, USA) and specific primers for each gene. Briefly, each reaction contained 10 μl, and the optimized cycling profile was performed at 95°C for 30 s, followed by 40 cycles at 95°C for 5 s, at 55°C for 34 s, and at 72°C for 45 s and the melting curve analysis at 95°C for 15 s and then at 60°C for 1 min. Each PCR was conducted in three technical triplicates. The *rpoS* gene (Ma et al., 2015) was used as reference, and positive and negative controls were included in all reaction mixtures.

### Cloning, Expression, and Purification of *V. parahaemolyticus* PMC53.7-*Zot* Gene in the *Escherichia coli* BL21 System

The full-length *V. parahaemolyticus* PMC53.7-*Zot* gene was amplified from the genomic DNA by PCR. The strains and plasmids used in this study are listed in Table 1. The amplified *Zot* gene was cloned into plasmid vector pBAD33.1 with 6-histidines tagged at the C-terminus and expressed following the manufacturer's instructions. The *E. coli* strain used for recombinant protein expression was BL21(DE3). The sequences of the primers used for *Zot* gene cloning are listed in Table 2. Vector control (pBAD33.1 without insert) was also subjected to identical treatment to that of *Zot* insert.

**Table 1:** Bacterial strains and plasmid used in this study.

**Bacterial strains**	**Relevant characteristics**	**Reference or source**
*V. parahaemolyticus* PMC 53.7	Clinical strain isolated from Puerto Montt, Chile	Laboratory collection
*E. coli* DH5α	F^−^*endA1 glnV44 thi-1 recA1 relA1 gyrA96 deoR nupG purB20* ϕ80d*lacZ*ΔM15 Δ(*lacZYA-argF*)U169, hsdR17(*r_*K*_*^−^*m_*K*_*^+^), λ^−^	Laboratory collection
*E. coli* BL21(DE3)	*E. coli* str. B F^−^*ompT gal dcm lon hsdS_*B*_*(*r_*B*_*^−^*m_*B*_*^−^) λ(DE3 [*lacI lacUV5*-*T7p07 ind1 sam7 nin5*]) [*malB*^+^]_K−12_(λ^S^)	Laboratory collection
**Plasmids**	**Relevant characteristics**	**Reference or source**
pBAD33.1	pBAD33 including ribosomal binding site, chloramphenicol resistant	pBAD33.1 was a gift from Christian Raetz (Addgene plasmid #36267)

*The amplified Zot gene was cloned into plasmid vector pBAD33.1 with 6-histidines tagged at the C-terminus and expressed following the manufacturer's instructions*.

**Table 2:** Sequences of primers used for *Zot* gene cloning.

**Primers**	Sequence (5′−3′)^*^	**Reference**
F1_pBAD33.1_ZotPMC53.7	*CTT*CATATG**GCTGTTATCTTTCGTCAC**	This study
R1_pBAD33.1_ZotPMC53.7	*AAC*AAGCTTtta*GTGGTGATGATGGTGATG***GCCCTCATTTAAGTTGAAAATATC**	

* *Extra base pairs on the 5′ end of each sequence denote primer leader (italicized); sequences with restriction sites of NdeI (forward) and HindIII (reverse) are underlined; Zot sequence residues are in boldface; tta represents the stop codon*.

The expression of Zot from BL21(DE3) pBAD33.1_ZotPMC53.7 was induced by the addition of 0.2% of l-arabinose at OD_600_ = 0.2 growth and harvested 3 h later to reach an OD_600_ = 0.5–0.6. The cells were harvested by centrifugation at 4,000 *g* for 20 min; resuspended in lysis buffer containing 50 mM Tris-HCl (pH = 7.4), 150 mM NaCl, Triton X-100 1%, 1 mM PSMF, 2% glycerol, lysozyme 1 mg/ml, and DNase I 10 μg/ml; and incubated on ice for 45 min before sonication. The mixture was sonicated with 24–25% amplitude for 5–7 min of total on time and 10–12 min of off time. The lysate was clarified by centrifugation, and the soluble fraction was used for purification of recombinant Zot protein. *V. parahaemolyticus* PMC53.7-Zot was purified by affinity-based purification using the nickel-IMAC resin HisPur Ni-NTA (Thermo Fisher Scientific, MA, USA) according to the manufacturer's instructions. As proteins eluted from the Ni-NTA columns contained both the PMC53.7-Zot and *E. coli* proteins, *E. coli* BL21(DE3) cells were transformed with pBAD33.1 vector without insert. The induction and purification of these proteins were performed using protocols identical to those for the purification of Zot. These *E. coli* proteins (EPs) were included as controls in all the experiments. The proteins eluted from Ni-NTA columns were filtered through 0.22 μm filters and concentrated through a buffer exchanged to DPBS using an Amicon Ultra 10K column (Merck Millipore Ltd, Carrigtwohill, Ireland). The total protein concentrations were determined using a Coomassie Plus (Bradford) assay kit (Thermo Fisher Scientific, MA, USA). The presence of *V. parahaemolyticus* PMC53.7-Zot was confirmed by SDS/PAGE followed by Coomassie staining and western blot analysis using anti-6xHis monoclonal mouse antibodies (Thermo Fisher Scientific, MA, USA).

### Actin Cytoskeletal Staining of Cells Incubated With Recombinant Zot Protein

The post-confluent Caco-2 cells were treated overnight with 25, 50, and 100 μg of PMC53.7-Zot or EP as control for actin cytoskeletal staining. Cells were washed three times with PBS without Ca^+2^ and Mg^+2^, fixed with PFA 4%, washed three times with PBS, and permeabilized with Triton X-100 0.1%. F-actin was stained using Alexa-Fluor 488 phalloidin (Thermo Fisher Scientific, MA, USA) at a dilution of 1:200 with PBS. Cells were subsequently rinsed, mounted, and viewed using an epifluorescence microscope (Leica LX6000, Germany). The mean pixel intensity value for actin was quantified using the NIH ImageJ software.

### Bacterial Toxin Prediction

The amino acid sequences of the Zot proteins from *V. parahaemolyticus* clinical strains PMC53.7 and VpKX (Q9KGQ7), *V. parahaemolyticus* environmental strains PMA2.15 and PMA3.15, *V. cholerae* N16961 (P38442), *C. concisus* 13826 (A7ZF54), and *Neisseria meningitidis* MC58 (Q9JY47) were obtained from UniProtKB. The toxin prediction from the primary amino acid sequence of Zot found in the *V. parahaemolyticus* clinical non-toxigenic strain PMC53.7, *V. cholerae* N16961, and other strains was performed using the BTXpred server (Saha and Raghava, 2007). This server uses SVM, HMM, and PSI-Blast to predict and classify exotoxins and endotoxins with an accuracy above 95%, besides identifying the function of enterotoxins with 100% overall accuracy.

### Multiple Sequence Alignment for Zot Proteins

The multiple sequence alignment (MSA) of the Zot amino acid sequences from *V. parahaemolyticus* PMC53.7, VpKX, PMA2.15 and PMA3.15, *V. cholerae* N16961 (VcN16961), *C. concisus* 13826 (Cc13826), and *N. meningitidis* MC58 (NmMC58) was performed with T-Coffee server and the PSI/TM-Coffee option (Notredame et al., 2000). The motifs and domains analyses were performed with the Conserved Domains Search tool (CD-Search) from NCBI (Marchler-Bauer et al., 2017). The figures were generated with Jalview (Waterhouse et al., 2009) and the WebLogo server (Crooks et al., 2004).

### Structure Prediction of *V. parahaemolyticus* PMC53.7-Zot Protein

The Protter (Omasits et al., 2014) and Phobius (Käll et al., 2004) servers were used for prediction of transmembrane topology of PMC53.7-Zot, as well as N-terminal and C-terminal domains. The potential phosphorylation sites were predicted with the NetPhos 3.1 server (Blom et al., 1999). Due to the lack of templates available in databases such as the Protein Data Bank (PDB), to perform conventional homology modeling, we modeled the 3D structure of PMC53.7-Zot with the I-TASSER server (Yang and Zhang, 2015). Residues 272–290 were preselected as the residues of the transmembrane segment, with α-helix as the predetermined secondary structure, according to the predictions made with the other servers to guide the modeling. The other I-TASSER parameters were set by default. Two threading templates were found and used by the server (PDB codes 2R2A and 3JC8) to finally generate five models. The top 1 model according to I-TASSER selection parameters (Zhang, 2008; Roy et al., 2010; Yang and Zhang, 2015) was validated with PROCHECK (Laskowski et al., 1993) and the ProSA-web server (Wiederstein and Sippl, 2007) and was selected for further modeling and analysis.

Model 1 was manually modified using the Maestro suite (Schrödinger Release 2019-3) to generate clear N-terminal (intracellular) and C-terminal (extracellular) domains as well as the transmembrane segment. Later, the protein was optimized and minimized using the Protein Preparation Wizard included in the Maestro Suite and subjected to two molecular dynamics simulations (MDs) using the Desmond MD package (Jorgensen et al., 1996) and the OPLS3 force field (Harder et al., 2016). The PMC53.7-Zot model was embedded into a 1-palmitoyl-2-oleoyl-phosphatidylcholine (POPC) preequilibrated membrane model (111 phospholipids per layer) and solvated with single-point charge (SPC) waters (57,315 molecules). The Cl^−^ ions were used as counterions in order to neutralize the systems, and 150 mM of NaCl was added to the system. For the first 25 ns, the default relax protocol of Desmond was applied. Then a restraint spring constant of 1 kcal ^*^ mol^−1^^*^^−2^ was applied to the backbone atoms of the protein. The last frame was taken, and a second non-restricted 250 ns MDs was performed. The temperature was maintained at 300 K, while pressure was kept at 1 atm, employing the Nosé–Hoover thermostat method with a relaxation time of 1 ps using the MTK algorithm (Martyna et al., 1994), with a 2 fs integration time step. Data were collected every 5 ps during the MDs for further analysis.

### Statistical Analysis

The values of LDH obtained in the cytotoxicity assay were analyzed with one-way ANOVA and a *post-hoc* Bonferroni test with 95% significance, using GraphPad Prism 6.0 software. The Zot data expression was analyzed using REST 2009 software (Pfaffl et al., 2002). The differences were considered statistically significant when ^*^p < 0.05, ^**^p < 0.01, and ^***^p < 0.001. The correlation analysis between the variables “Zot mRNA expression” and “cytotoxicity” was determined using Pearson correlation analysis, and it was interpreted that a value ≥0.7 indicates a significant and positive relationship between both variables (Nettleton, 2014).

## Results

### Contribution of Zot-PMC53.7 to Cytotoxicity in Caco-2 Cells

**Figure 1:**
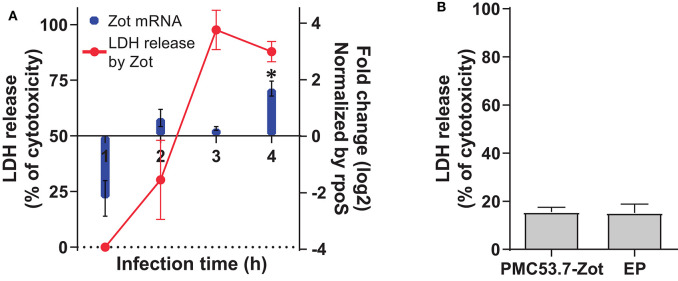
Transcriptional level of *Zot* mRNA during course of infection and cytotoxicity. **(A)** Zot expression levels of PMC53.7 strain and cytotoxicity (%) during the course of infection of Caco-2 cells. * Up-regulated vs rpoS (reference gene) **(B)** Cytotoxicity (%) of Caco-2 cells induced by treatment with 100 μg of purified PMC53.7-Zot and *E. coli* proteins (EP) control. All the experiments were done in triplicate and repeated three times.

In our previous work, we identified Zot-encoding genes in the genomes of highly cytotoxic Chilean *V. parahaemolyticus* strains (Castillo et al., 2018a). Then, we hypothesized that PMC53.7-Zot could contribute to cytotoxicity. To assess whether Zot expression occurred during *V. parahaemolyticus* PMC53.7 infection of Caco-2 cells, a kinetic infection was performed for 4 h. The analysis of gene *Zot* by qPCR showed detectable levels of expression after 2 h post infection and a gene overexpression at 4 h post infection, relative to the reference gene *rpoS* (Figure 1A). In parallel, we evaluated if Zot mRNA levels could be correlated with the cytotoxicity induced by *V. parahaemolyticus* PMC53.7. The LDH release was measured at each time point during PMC53.7 infection kinetics of Caco-2 cells (Figure 1A). Pearson analysis showed that there was a barely positive correlation between Zot mRNA expression and LDH release, with a global correlation coefficient of 0.7 (Nettleton, 2014). Pearson correlation coefficients for each independent experiment were 0.87, 0.61, and 0.76. PMC53.7-Zot, previously expressed in a heterologous system of *E. coli* BL21, was purified and was visualized by western blot as a band of ~57 kDa, according to the fully transcribed *Zot* gene (56 kDa), while a second band of ~27 kDa was observed (Supplementary Figure 1). Unexpectedly, Caco-2 cells treated with purified PMC53.7-Zot (100 μg) did not exhibit cytotoxicity (Figure 1B); instead, we observed that they were impaired to be attached to the plate surface at 4 h post treatment, which was not detected in the control treatment with *E. coli* proteins (EP) (Supplementary Figure 2).

### Cellular Damage Provoked by Infection With PMC53.7

**Figure 2:**
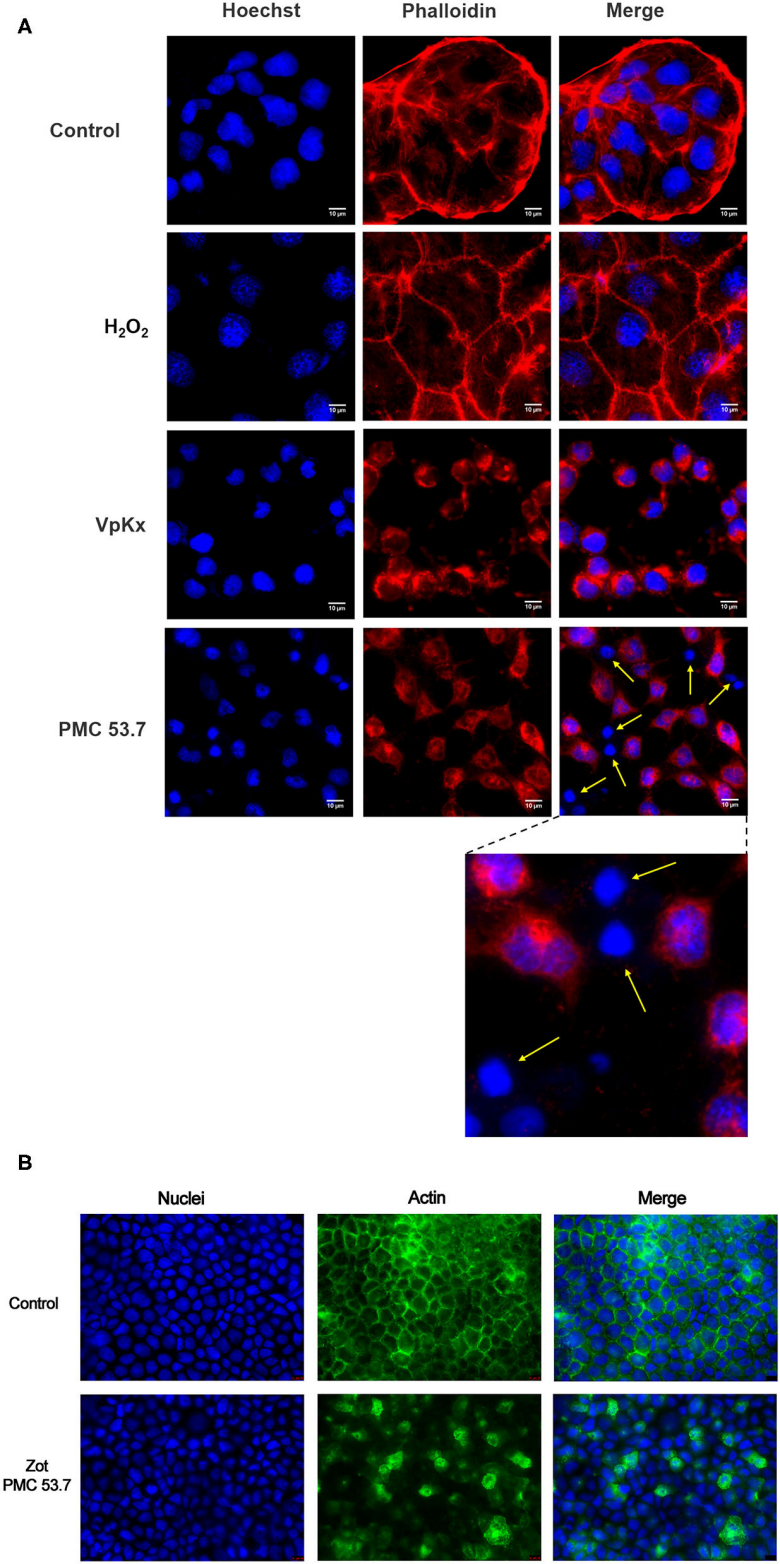
Effect of PMC53.7 infection over the morphology of Caco-2 cells. **(A)** PMC53.7 infection (MOI 10) produces disruption of the actin cytoskeleton in infected cells at 3 h post infection. The yellow arrows indicate the absence of actin in several cells infected with PMC53.7. **(B)** Caco-2 exposed to 100 μg of purified PMC53.7-Zot showed an increased percentage (at 24 h incubation) of cells displaying redistribution of F-actin compared to the control-exposed cells.

It is well-known that the main contributor of cytotoxicity in *V. parahaemolyticus* is the T3SS-1, which is completely present in PMC53.7 as also in VpKX strains. If Zot was not contributing to cytotoxicity over T3SS1, we hypothesized that detrimental effects exclusively displayed by PMC53.7 infection could be associated with its unique putative virulence factor Zot, in the absence of TDH, TRH, and T3SS-2. In parallel to cytotoxicity assays, we decided to compare effects of both strains over the cell culture. We infected Caco-2 cells with *V. parahaemolyticus* PMC53.7 and VpKX at an MOI = 10 and performed fluorescence microscopy at 3 h post infection. We observed that uninfected monolayers of Caco-2 cells had an organized actin cytoskeleton in a network of filaments normally distributed beneath the plasma membrane and throughout the cytoplasm (Figure 2A). On the other hand, infected cells showed cytoskeletal rearrangement and detachment of adjacent cells from each other with PMC53.7 and VpKX (Figure 2A). Interestingly, we observed the absence of actin in several cells infected with PMC53.7 (yellow arrows, Figure 2A), which was not observed in the VpKX infection. As an additional control of cellular damage, we treated Caco-2 cells with 1 mM H_2_O_2_, but its effects over cellular nuclei were not observed in the infected cells (Figure 2A). In addition, we visualized the actin cytoskeleton of Caco-2 incubated with PMC53.7-Zot, through immunofluorescence with fluorescent phalloidin. These cells showed a higher percentage of F-actin redistribution, compared to control cells, with a peak at 24 h of incubation. Both Caco-2 control and EP incubated cells had stabilized F-actin with normal, continuous, and smooth distribution of actin at the membrane boundaries (Figure 2B). Instead, the treatment with 100 μg of PMC53.7-Zot produced rearrangement of actin in the cells (Figure 2B). These results suggest that Zot could play a key role in *V. parahaemolyticus* PMC53.7 infection, inducing the loss of actin cytoskeleton integrity in Caco-2 cells (Figure 2B).

### Bioinformatics Analysis

Since we observed that PMC53.7 infection produces disruption of the actin cytoskeleton in infected cells, which is an effect associated to the Zot action of *V. cholerae* over IEC6 cellular culture (Fasano et al., 1995), we decided to perform a bioinformatics analysis, comparing the sequence of *V. parahaemolyticus* PMC53.7-Zot with Zot sequences of other important human pathogens: *V. cholerae* N16961 (VcN16961) and *C. concisus* 13826 (Cc13826), which have been reported as biologically active toxins (Fasano et al., 1995; Mahendran et al., 2016); and we also included *N. meningitidis* MC58 (NmMC58) and other strains of *V. parahaemolyticus* (PMA2.15, PMA3.15, and the reference strain VpKX).

### Prediction of *V. parahaemolyticus* PMC53.7-Zot as Toxin

To investigate if the Zot amino acid sequence found in PMC53.7 was a toxin, we performed a comparison of this sequence against known toxin databases using BTXpred (Saha and Raghava, 2007). As control, we used the Zot sequences of VcN16961 and Cc13826. The results showed that PMC53.7-Zot and VcN16961-Zot were recognized as endotoxin, while Cc13826-Zot matched with an exotoxin (Table 3). Additionally, we performed the same analysis with Zot sequences of other *V. parahaemolyticus* strains. We observed that VpKX-Zot and PMA2.15-Zot were classified as exotoxin with a guanylate cyclase-activating enterotoxin function, while PMA3.15-Zot and NmMC58-Zot did not match with any toxin (Table 3).

**Table 3:** Bacterial toxin prediction using BTXpred.

**Species**	**Strain**	**Toxin classification**	**Exotoxin function**
*V. parahaemolyticus*	PMC53.7	Endotoxin	–
*V. parahaemolyticus*	VpKX	Exotoxin	Guanylate cyclase activating enterotoxin
*V. parahaemolyticus*	PMA2.15	Exotoxin	Guanylate cyclase activating enterotoxin
*V. parahaemolyticus*	PMA3.15	Not match	–
*V. cholerae*	N16961	Endotoxin	–
*C. concisus*	13826	Exotoxin	Not found
*N. meningitidis*	MC58	Not match	–

*We used the Zot amino acid sequences of V. parahaemolyticus VpKX, PMC53.7, PMA2.15, and PMA3.15; V. cholerae VcN16961; C. concisus Cc13826; and N. meningitidis MC58, comparing each sequence against known toxin databases using the BTXpred program (Saha and Raghava, 2007)*.

### MSA in Different Zot Proteins and Their Walker A and Walker B Motifs

**Figure 3:**
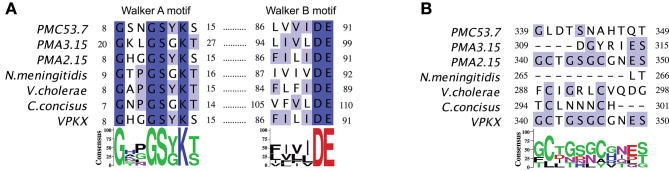
Multiple sequence alignment of Zot protein. **(A)** Conserved Walker A (GxxxxGK[S/T]) and B (hhhh[D/E]) motifs. **(B)** Zot-active domain and putative Zot-receptor-binding site proposed to V. cholerae N16961.

To detect conserved domains and motifs present in the Zot protein sequences of *V. parahaemolyticus*, an MSA was performed comparing Zot of different species of human pathogens, including strains previously mentioned (Supplementary Figure 3). A detailed analysis of the protein sequences showed that these proteins belong to the P-loop containing nucleoside triphosphate hydrolases. Members of the P-loop NTPase domain superfamily are characterized by a conserved nucleotide phosphate-binding motif, also referred as the Walker A motif (GxxxxGK[S/T], where x is any residue), and the Walker B motif (hhhh[D/E], where *h* is a hydrophobic residue) (Hanson and Whiteheart, 2005). Respect to PMC53.7, the protein sequence identity of NmMC58, VcN16961, Cc13826, and VpKX/PMA2.15 is 30.9, 23.8, 38.5, and 40.7%, respectively. We noticed that Zot sequences of *V. parahaemolyticus* strains, including both clinical PMC53.7 and VpKX and environmental strains PMA 2.15 and PMA 3.15, besides VcN16961, have a tyrosine (Y) instead of a glycine (G) in the Walker A motif: GxxxxYK[S/T] (Figure 3A). Both Walker motifs were located at the N-terminal side prior to the transmembrane domains, approximately 1–270, as defined for *V. cholerae*-Zot (Uzzau et al., 1999). As these Walker motifs belong to the proteins of the P-loop NTPase superfamily, we aligned the sequence of *V. parahaemolyticus* PMC53.7 against the protein sequence of PHA00350 (Supplementary Figure 4), member of the P-loop NTPase superfamily (conserved protein domain family accession number: cl21455).

No sequences of the Zot proteins in *V. parahaemolyticus* isolates had the FCIGRL active fragment previously identified in *V. cholerae* and located in the C-terminal domain (Goldblum et al., 2011), neither the Zot sequences of *C. concisus* nor *N. meningitidis* (Figure 3B). Additionally, we noticed that PMC53.7, *C. concisus, N. meningitidis*, and VpKX do not have a glycine aligned with the *V. cholerae* position 298, which has been proposed as a key amino acid involved in the opening of the intercellular tight junctions (TJ) (Figure 3B). Instead, PMC53.7 and *N. meningitidis* have a threonine residue, while VpKX, PMA2.15, and PMA3.15 have a serine in this position. This G-298 position is the last amino acid of an octapeptide motif (GxxxVQxG) proposed as the putative receptor-binding site shared by Zot and human zonulin (Di Pierro et al., 2001). The octapeptide motif was not found in any other pathogenic bacterial strains besides *V. cholerae* (Figure 3B).

### Structure Prediction of *V. parahaemolyticus* Zot Proteins

Besides the prediction of *V. parahaemolyticus* PMC53.7-Zot as an endotoxin, similar to *V. cholerae*-Zot, we focused on predicting its structure, because the *Zot*-coding gene was the unique putative virulence factor found in the genome of this clinical strain (Castillo et al., 2018a). In addition, it has been suggested that the structure and not the sequence is responsible for the biological effects of Zot on the epithelial barrier (Kaakoush et al., 2010), and also cytoskeletal disturbances occurred in response to *V. cholerae*-Zot (Fasano et al., 1995). So, despite some differences reported among *V. parahaemolyticus*-Zot and *V. cholerae*-Zot sequences, we decided to perform 3D structure prediction of PMC53.7-Zot. First, we predicted a transmembrane domain of PMC53.7-Zot (Supplementary Figures 5A–D), as *V. cholerae*-Zot has been reported as a transmembrane protein. Three well-defined domains were identified for PMC53.7-Zot as follows: an N-terminus from residues 1 to 272; one transmembrane segment from 273 to 294; and a C-terminus from 295 to 466. We also predicted the phosphorylation sites in the PMC53.7-Zot (above the threshold value in Supplementary Figure 5B using NetPhos 3.1 server; Blom et al., 1999). Specifically, the PMC53.7-Zot was predicted to possess 23 serine-, 21 threonine-, and 6 tyrosine-phosphorylation sites, all of them equally distributed at the N-terminal and C-terminal domains. Finally, properties such as solvent accessible surface area (SASA), hydropathy, and the inherent thermal mobility of the residues/atoms in PMC53.7 protein were also predicted (Supplementary Figures 5E–G).

The I-TASSER server was found, by threading two suitable templates, to generate multiple PMC53.7-Zot models (PDB codes: 2R2A and 3JC8). The software used the templates 2R2A and 3JC8 to model the PMC53.7-Zot N-terminal and C-terminal domains, respectively. PMC53.7-Zot shared 25.71% of sequence identity with 35% of coverage to 2R2A (chain A) and 60% of sequence identity with 10% of coverage to 3JC8 (chain Q). The coverage is the number of aligned residues of each template divided by the length of PMC53.7-Zot; in both cases, the coverage is very low. The I-TASSER server generated an MSA with both template sequences (Supplementary Figure 6) and then a large ensemble of structural conformations. The top five models (according to I-TASSER scoring function) generated were further analyzed (Supplementary Figure 7). It is clear that the only valid template to generate a valid model is 2R2A; therefore, in this work, we assume that the structure of the N-terminal domain of PMC53.7-Zot modeled using 2R2A as a template is reliable. This can be corroborated according to the I-TASSER estimated accuracy (the lower, the better) of models 1 to 5, where residues of the C-terminal domain present higher estimated accuracy than the transmembrane segment as well as the N-terminal domain (Supplementary Figure 7).

**Figure 4:**
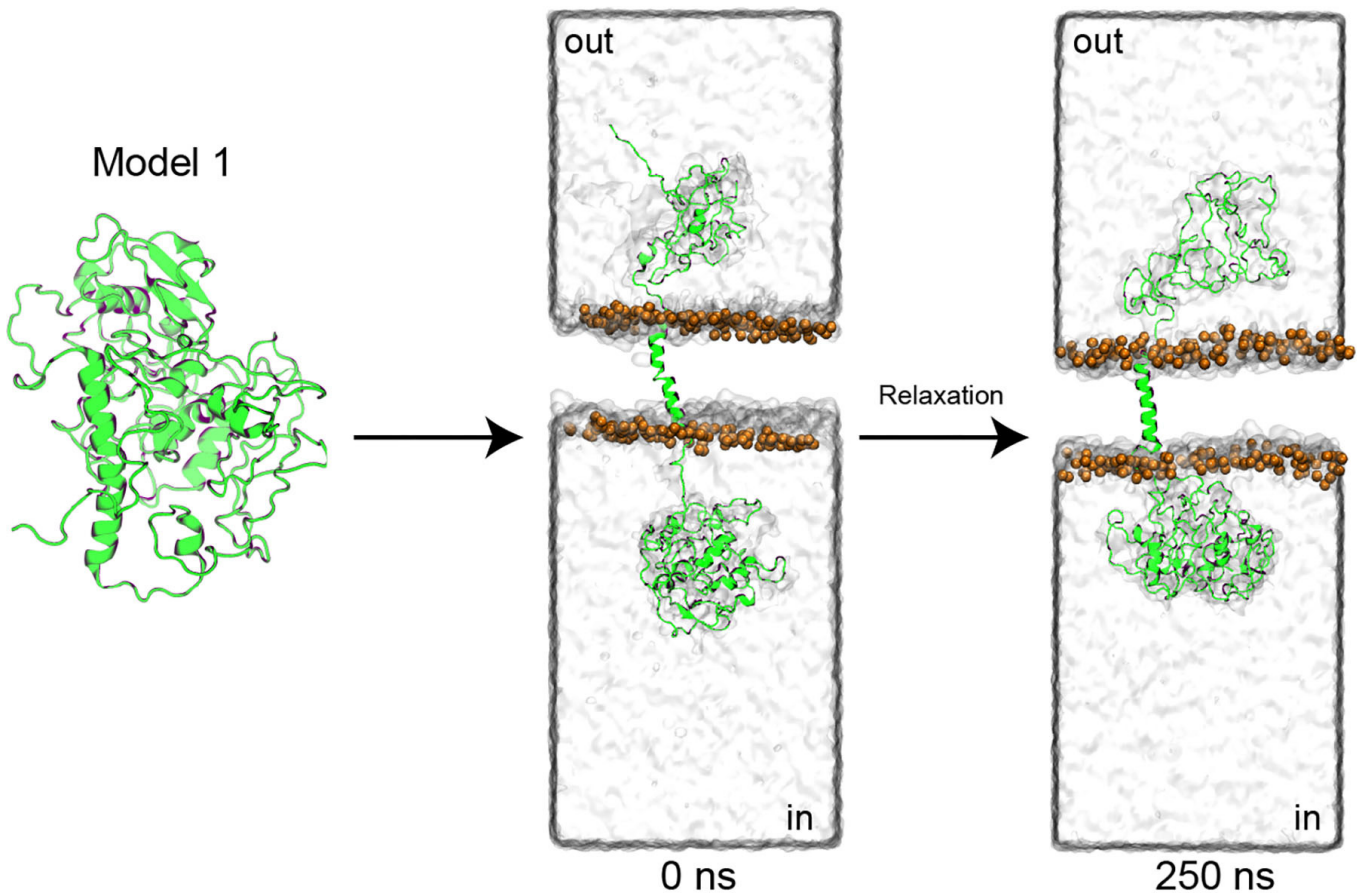
PMC53.7-Zot model refinement. The top-1 PMC53.7 model generated by I-TASSER was manually modified to produce the N-terminal and C-terminal domains at the intracellular and extracellular compartments, respectively. The protein is shown as a cartoon representation; the solvent is displayed as a water-surface box; only the phosphates atoms from the POPC membrane are displayed as orange spheres for better visualization.

Then, model 1 was selected and manually modified to generate clear N-terminal (cytoplasmic) and C-terminal (extracellular) domains (Figure 4), as well as the transmembrane segment, previously predicted (Supplementary Figure 5). After the generation of clear domains, the model was embedded into a POPC membrane (111 phospholipids per layer) and solvated with water (57.315 molecules). Later, the system was subjected to two molecular dynamics simulations (MDs). The first 25 ns of simulation was performed with application of a restraint spring constant of 1 kcal ^*^ mol^−1^^*^^−2^ to the backbone atoms of the protein; then, the last frame was taken, and a second non-restricted 250 ns MDs was performed (Figure 4).

The root mean square deviation (RMSD) of the backbone atoms, as a function of simulation time, was analyzed to know how stable the model was after the 250 ns MDs, using their initial configuration (0 ns) as reference (Supplementary Figure 8). It is possible to observe that the protein is not stable because the global RMSD is around 10–20 Å after 20 ns, due to the mobility of the C-terminal and N-terminal domains. When the three different domains were analyzed separately, we observed that the transmembrane segment was very stable (RMSD <5 Å), followed by the N-terminal (RMSD <10 Å) and C-terminal (RMSD <20 Å) domains. This allows us to evidence that the N-terminal domain was indeed well-modeled and that it is stable over time, as well as the transmembrane segment. The quality of the C-terminal domain cannot be verified due to the fact that it was modeled without a suitable template. To characterize changes in the PMC53.7-Zot residue position along the 250 ns unrestrained MDs of our final model, the root mean square fluctuation (RMSF) was calculated, showing the mobility of the protein residues along the MDs (Supplementary Figure 8C). The major fluctuations were identified in the C-terminal domain. This is in agreement with the stable time dependence of RMSD for the N-terminal domain as well as the transmembrane segment in our model, and it indicates that the major rearrangement of its conformation during the MDs occurs at the C-terminal domain, as expected. The model was validated using PROCHECK (Laskowski et al., 1993) and ProSA (Wiederstein and Sippl, 2007). The validations were done with the initial model 1 obtained from I-TASSER, the model before (0 ns) and after (250 ns) MDs, and finally with the model without the C-terminal domain (Supplementary Figure 9), because (as stated above) this region was modeled with an unsuitable template (low residue identity %). This result indicated that the model was reasonable for the transmembrane segment and N-terminal domain. Furthermore, the validations showed that as the model is refined, the quality of the 3D structure modeled improves. While the model does not show residues in the more favorable regions above 75% of the Ramachandran plots (Bertini et al., 2003) (Supplementary Figure 9), it is clear that residues in disallowed regions begin to shift to allowed regions as the model is refined through MDs. The final quality of the model indicates that the C-terminal and N-terminal parts are quite mobile and that as the model is refined, the number of residues in permitted regions increases. To obtain a better-quality 3D structure, it is necessary to find templates with higher percentages of sequence identity and coverage, but unfortunately, in our case, we do not have templates with these characteristics. The *z*-score of the stabilized model after 250 ns is in the same range as the experimentally determined structure proteins of the PDB (Wiederstein and Sippl, 2007) (Supplementary Figure 10).

Finally, we monitored the protein secondary structure elements (SSE) like α-helices and β-strands during the simulation. Supplementary Figure 11A shows the percentage of SSE distribution by residue index throughout the protein structure. We found that out of 100% of the PMC53.7-Zot model, 8.82% corresponds to α-helix and 2.25% to β-strands (total of SSE assigned = 11.41%). Supplementary Figure 11B summarizes each residue, and its SSE assignment over the 250 ns MDs. And the 3D structure of the equilibrated model (at 250 ns) is displayed with α-helices, β-strands, and loops highlighted in Supplementary Figure 11C. The most stable secondary structure is the transmembrane segment, followed by the α-helices and β-strands at the N-terminal domain. These results are in concordance with the initial prediction of the secondary structure of the protein (Supplementary Figure 5).

## Discussion

The present study identifies and characterizes novel virulence factors that could explain the pathogenicity of non-toxigenic strains of *V. parahaemolyticus*. In our previous work, we identified that some of these strains possess *Zot* genes in their accessory genome associated with prophages (Castillo et al., 2018a). The studies across diverse marine *Vibrio* species have shown that filamentous prophages play a key role in the emergence of novel pathogenic strains from the environment (Hay and Lithgow, 2019). In addition, we showed that the identification of Zot occurred exclusively in highly cytotoxic strains (Castillo et al., 2018a), suggesting a possible role for *V. parahaemolyticus*-Zot. In this work, we observed that there was a barely positive correlation between *Zot* mRNA expression occurring during *V. parahaemolyticus* PMC53.7 infection of Caco-2 cells and cellular membrane damage represented by LDH release (% of cytotoxicity). Additionally, the treatment of Caco-2 cells with purified PMC53.7-Zot heterologous produced in *E. coli* BL21 did not induce cytotoxicity. Although the main band observed by Western blot corresponded to the expected size for the complete transcribed *Zot* gene (56 kDa), a second band of ~27 kDa was observed (Supplementary Figure 1). The significance of the second one is unknown, but the *V. cholerae*-Zot undergoes a proteolytic cleavage after the transmembrane domain, which releases the biologically active C-terminal fragment (12 kDa) into the intestinal micro milieu (Goldblum et al., 2011). We cannot affirm whether the two bands obtained for PMC53.7-Zot occurred due to the action of bacterial proteases in the *E. coli* host or by autoproteolysis. However, the ~27 kDa of the second band is close to the predicted size of the C-terminal fragment (22 kDa, Supplementary Figure 1), plus the histidine tail (4.5 kDa). This suggests that there probably exists a sequence site that favors the PMC53.7-Zot cleavage after the transmembrane domain. Nonetheless, the absence of correlation among Zot and cytotoxicity observed to PMC53.7-Zot had been previously reported to *V. cholerae*-Zot (Fasano et al., 1995). Interestingly, despite not finding a strong cytotoxicity correlation, we observed that PMC53.7-Zot impaired the attachment of Caco-2 cells to the plate surface (Supplementary Figure 2), suggesting disturbance of focal adhesions. In addition, we also observed alterations of actin cytoskeleton associated with the infection with PMC53.7, which were not observed with VpKX (Figure 2A) and actin rearrangements in response to the protein treatment (Figure 2B). Based on both observations, we suggest that probably *V. parahaemolyticus* PMC53.7-Zot contributes to cause redistribution of actin cytoskeleton also described for the *V. cholerae*-Zot. The effect observed in Caco-2 cells could be explained by differences in the actin distribution, since it is the F-actin cytoskeleton, as well as its connection to the plasma membrane, that is responsible for providing the structure and shape of epithelial cells (Brückner et al., 2019). Regrettably, despite all efforts to obtain a PMC53.7-ΔZot strain, it was not possible, even using diverse methodologies. However, the similar effects observed in Caco-2 cells infected with PMC53.7 (Figure 2A) and treated with PMC53.7-Zot (Figure 2B) suggest that the actin cytoskeleton alterations observed exclusively during the infection with PMC53.7 (yellow arrows in Figure 2A) occurred due to the Zot action. On the other hand, although the H_2_O_2_ effects over cellular nuclei were not detected in any of the infected cells, the nucleus fragmentation observed with Hoechst staining and the cytoskeletal alteration detected with phalloidin in Caco-2 cells infected with PMC53.7 suggest preliminarily that it could be a type of death related to apoptosis; however, the result did not support by itself that conclusion, and additional experiments should be done to elucidate the type of cell death induced by the *V. parahaemolyticus* PMC53.7 strain. Future research will address the unanswered aspects of this study.

It would be expected that similar functions are being attributed to similar domains of Zot protein. In fact, the bioinformatics analysis showed several similarities between Zots of *Vibrio* pathogens. The N-terminal region of PMC53.7-Zot was highly conserved among *Vibrio* strains, *Vibrio* species, and other human pathogenic bacterial species which possess Zot associated with prophages in its accessory genomes. The unique highly conserved protein among the filamentous phages is the pI, which has a conserved Zot domain (Pfam PF05707) at the N-terminus. This domain is essential for the assembly and export of phage virion, and it was named for the homolog in the *Vibrio* CTX phage (Hay and Lithgow, 2019). We also identified two Walker motifs located toward the N-terminal region, prior to the transmembrane domain of *V. parahaemolyticus*-Zot. Walker A and B motifs belong to the proteins of the P-loop NTPase superfamily (Hanson and Whiteheart, 2005). In fact, the sequence of *V. parahaemolyticus* PMC53.7-Zot aligned against the protein sequence of PHA00350, a putative assembly protein, which is a member of the P-loop_NTPase superfamily (accession number: cl21455). The P-loop_NTPase binds to NTP, typically ATP or GTP, through the Walker A and B motifs. Specifically, the N-terminus of Zot is predicted to act as an ATPase, powering the assembly and transport of phages through the envelope, as has been observed for *E. coli* Ff-type phages (Feng et al., 1997). It has been identified that P-loop in NTPases is able to affect focal adhesion and actin fibers of cells (Steele-Mortimer et al., 2000); thus, there exists the possibility that the conserved motifs located toward the N-terminal end of the toxin could be responsible for the attachment impairment seen after PMC53.7-Zot treatment in the cells (Supplementary Figure 2). Besides, a change of glycine (non-polar aliphatic amino acid) to tyrosine (aromatic amino acid) into the Walker A motif (GxxxxGK[S/T]) observed in most *V. parahaemolyticus* strains was also observed in *V. cholerae* (GxxxxYK[S/T]) but did not occur in *N. meningitidis* and other *Campylobacter* species (Liu et al., 2016). Despite this change, the *V. cholerae*-Zot maintains the functionality (Schmidt et al., 2007).

The mechanism of action of *V. cholerae*-Zot has been deeply studied, and it is known that it depends on the active fragment FCIGRL and its binding to the zonulin receptor PAR-2 (Goldblum et al., 2011). However, FCIGRL is absent in PMC53.7-Zot, any *V. parahaemolyticus* strain contained the active fragment described for *V. cholerae* in its Zot sequences, and there was a high variability on the C-terminal end of Zot between different pathogens and among *Vibrio* species and strains. Interestingly, we observed that differences of these regions were responsible for the diversity between Zot sequences of *V. parahaemolyticus* strains. In fact, three Zot sequences of PMC53.7, PMA2.15, and PMA3.15 strains were classified into three categories using BTXpred: endotoxin, exotoxin with guanylate cyclase activating enterotoxin activity, and non-toxin, respectively. Likewise, PMA3.15 was less cytotoxic on Caco-2 cells than other non-toxigenic strains (see Figure 7 in Castillo et al., 2018a). On the other hand, VcN16961-Zot was classified as endotoxin while Cc13826-Zot was identified as an exotoxin with an unknown activity. The diversity among Zot sequences, found in diverse *Vibrio* species, was suggested in our previous work. The phylogenetic analysis of different toxin sequences showed that *V. parahaemolyticus* PMA2.15-Zot was identical to that found in the phage f237 of VpKX, while *V. parahaemolyticus* PMC53.7-Zot was grouped, in other nearby clades, with *V. parahaemolyticus*_A0A1J0JZE6, *Vibrio campbellii*, and *V. parahaemolyticus*_A0A0N1IWZ0 strains. Interestingly, PMA3.15-Zot, not recognized as a toxin in this work, was the most divergent sequence, and it was grouped with *Vibrio celticus* in a distant clade, suggesting major variability. This Zot divergence between different clades was observed for *V. campbellii* and *Vibrio splendidus*; however, Zot of *V. cholerae* had a major similarity among them, and all sequences were grouped in only one clade (see Figure 5 in Castillo et al., 2018a). If the diversity of Zot sequences can have an impact on its definition as toxin, we would expect that Zot of *V. cholerae* strains would act as endotoxins, but not all Zots of *V. parahaemolyticus* would have the same mechanism of action. Even more, not all Zots found in *V. parahaemolyticus* should be considered as active toxins; thus, Zot sequences with the ability to produce detrimental effects over human cells must be clearly recognized and subsequently detected.

The absence of the FCIGRL fragment is also observed in the Zot sequence of *C. concisus*, which is able to affect the paracellular pathway in spite of its absence (Mahendran et al., 2016), suggesting that the presence of this peptide sequence would not be strictly necessary to perform the action of all Zots. Similarly, a glycine in position 298 of *V. cholerae*-Zot, with a proposed crucial role in the opening of intracellular TJ, was also absent in all *V. parahaemolyticus* and *C. concisus* strains. The above observations are related to those previously reported by Kaakoush et al. (2010), so it would be the structure and not the sequence that is responsible for the biological effects of Zot on the epithelial barrier. For this reason, we modeled the PMC53.7-Zot sequence. The structure prediction suggested the presence of a transmembrane helix, which would allow PMC53.7-Zot to be specifically anchored to the membrane, as also has been reported to *V. cholerae* (Di Pierro et al., 2001). Considering that Zot could be responsible for cellular actin disturbances in Caco-2 cells, we suggest that the high number of phosphorylation sites could constitute a mechanism for regulation of protein secretion. In this regard, we reported a model for PMC57.3-Zot with the aim of contributing a structural approach to understand the function of this protein. The prediction and refinement of the structural model of the Zot protein, carried out in this work, show that a relatively stable model can be established for the N-terminal and transmembrane domains of the protein. However, it was not possible to obtain a reliable prediction for the C-terminal domain because there is no suitable model. Despite a molecular dynamic of 250 ns for the whole structure, the C-terminal domain showed high fluctuations between 10 and 20 Å. We hope that this partially stable structural model of Zot will contribute to future research to elucidate its function as a possible virulence determinant.

In conclusion, our results show that PMC53.7-Zot cannot induce cytotoxicity in Caco-2 cells, as we previously suspected. Instead, we suggest that it would be responsible for the actin cytoskeletal disturbance in the infected cells, as also described for *V. cholerae*-Zot (Goldblum et al., 2011). However, whether this effect is due to the conserved NTPase activity of the N-terminus, the 3D structural similarity with the *V. cholerae*-Zot, or a combination of both is a matter of future studies. Furthermore, the present study offers the entire model of PMC57.3-Zot as we consider it important to highlight that there are no suitable templates to model all the domains. However, a good approach to understanding the function of this protein through its structure can be made by following a rigorous modeling process.

## Data Availability Statement

All datasets generated for this study are included in the article/Supplementary Material.

## Author Contributions

KG and DP-R conceived the idea. KG, DR, CB, DP-R, and AP designed the experiments and wrote the manuscript. DR, CP-V, NP, MA-A, and CB performed the bioinformatics analysis and structure prediction. SR-A, VJ, LP, and AP performed infection and staining analysis. DP-R and RB performed Zot cloning, expression, and purification experiments. CL-J, GC, AP, and TP performed time course infection for cytotoxicity measures and made the statistical analysis. Fluorescence microscopy was performed by DP-R and SR-A. All the authors read, discussed, and approved the final version of this manuscript.

## Conflict of Interest

The authors declare that the research was conducted in the absence of any commercial or financial relationships that could be construed as a potential conflict of interest.

## References

[B1] Bertini I. Cavallaro G. Luchinat C. Poli I. (2003). A use of Ramachandran potentials in protein solution structure determinations. J. Biomol. NMR. 26, 355–366. 10.1023/A:1024092421649 12815262

[B2] Blom N. Gammeltoft S. Brunak S. (1999). Sequence and structure-based prediction of eukaryotic protein phosphorylation sites. J. Mol. Biol. 294, 1351–1362. 10.1006/jmbi.1999.3310 10600390

[B3] Broberg C. A. Calder T. J. Orth K. (2011). Vibrio parahaemolyticus cell biology and pathogenicity determinants. Microbes Infect. 13, 992–1001. 10.1016/j.micinf.2011.06.013 21782964 PMC3384537

[B4] Brückner B. R. Nöding H. Skamrahl M. Janshoff A. (2019). Mechanical and morphological response of confluent epithelial cell layers to reinforcement and dissolution of the F-actin cytoskeleton. Prog. Biophys. Mol. Biol. 144, 77–90. 10.1016/j.pbiomolbio.2018.08.010 30197289

[B5] Castillo D. Kauffman K. Hussain F. Kalatzis P. Rørbo N. Polz M. F.. (2018b). Widespread distribution of prophage-encoded virulence factors in marine Vibrio communities. Sci. Rep. 8:9973. 10.1038/s41598-018-28326-9 29967440 PMC6028584

[B6] Castillo D. Pérez-Reytor D. Plaza N. Ramírez-Araya S. Blondel C. J. Corsini G.. (2018a). Exploring the genomic traits of non-toxigenic Vibrio parahaemolyticus strains isolated in southern Chile. Front. Microbiol. 9:161. 10.3389/fmicb.2018.00161 29472910 PMC5809470

[B7] Ceccarelli D. Hasan N. A. Huq A. Colwell R. R. (2013). Distribution and dynamics of epidemic and pandemic Vibrio parahaemolyticus virulence factors. Front. Cell Infect. Microbiol. 3:97. 10.3389/fcimb.2013.00097 24377090 PMC3858888

[B8] Crooks G. E. Hon G. Chandonia J. M. Brenner S. E. (2004). WebLogo: a sequence logo generator. Genome Res. 14, 1188–1190. 10.1101/gr.849004 15173120 PMC419797

[B9] Di Pierro M. Lu R. Uzzau S. Wang W. Margaretten K. Pazzani C.. (2001). Zonula occludens toxin structure-function analysis. J. Biol. Chem. 276, 19160–19165. 10.1074/jbc.M009674200 11278543

[B10] Fasano A. (2002). Toxins and the gut: role in human disease. Gut 50(Suppl. III), iii9–iii14. 10.1136/gut.50.suppl_3.iii9 PMC1867677 11953326

[B11] Fasano A. Fiorentini C. Donelli G. Uzzau S. Kaper J. B. Margaretten K.. (1995). Zonula occludens toxin modulates tight junctions through protein kinase C-dependent actin reorganization, *in vitro*. J. Clin. Invest. 96, 710–720. 10.1172/JCI118114 7635964 PMC185254

[B12] Feng J. N. Russel M. Model P. (1997). A permeabilized cell system that assembles filamentous bacteriophage. Proc. Natl. Acad. Sci. U.S.A. 94, 4068–4073. 10.1073/pnas.94.8.4068 9108106 PMC20569

[B13] Fuenzalida L. Hernández C. Toro J. Rioseco M. L. Romero J. Espejo R. T. (2006). Vibrio parahaemolyticus in shellfish and clinical samples during two large epidemics of diarrhoea in southern Chile. Environ. Microbiol. 8, 675–683. 10.1111/j.1462-2920.2005.00946.x 16584479

[B14] Goldblum S. E. Rai U. Tripathi A. Thakar M. De Leo L. Di Toro N.. (2011). The active Zot domain (aa 288-293) increases ZO-1 and myosin 1C serine/threonine phosphorylation, alters interaction between ZO-1 and its binding partners, and induces tight junction disassembly through proteinase activated receptor 2 activation. FASEB J. 25, 144–158. 10.1096/fj.10-158972 20852064 PMC3005425

[B15] Gopalakrishnan S. Pandey N. Tamiz A. P. Vere J. Carrasco R. Somerville R.. (2009). Mechanism of action of ZOT-derived peptide AT-1002, a tight junction regulator and absorption enhancer. Int. J. Pharm. 365, 121–130. 10.1016/j.ijpharm.2008.08.047 18832018

[B16] Hanson P. I. Whiteheart S. W. (2005). AAA+ proteins: have engine, will work. Nat. Rev. Mol. Cell Biol. 6, 519–529. 10.1038/nrm1684 16072036

[B17] Harder E. Damm W. Maple J. Wu C. Reboul M. Xiang J. Y.. (2016). OPLS3: a force field providing broad coverage of drug-like small molecules and proteins. J. Chem. Theory Comput. 12, 281–296. 10.1021/acs.jctc.5b00864 26584231

[B18] Harth E. Matsuda L. Hernández C. Rioseco M. L. Romero J. González-Escalona N.. (2009). Epidemiology of Vibrio parahaemolyticus outbreaks, Southern Chile. Emerg. Infect. Dis. 15, 163–168. 10.3201/eid1502.071269 19193258 PMC2657608

[B19] Hay I. D. Lithgow T. (2019). Filamentous phages: masters of a microbial sharing economy. EMBO Rep. 20:e47427. 10.15252/embr.201847427 30952693 PMC6549030

[B20] Hidalgo I. J. Raub T. J. Borchardt R. T. (1989). Characterization of the human colon carcinoma cell line (Caco-2) as a model system for intestinal epithelial permeability. Gastroenterology 96, 736–749. 10.1016/S0016-5085(89)80072-1 2914637

[B21] Jorgensen W. L. Maxwell D. S. Tirado-Rives J. (1996). Development and testing of the OPLS all-atom force field on conformational energetics and properties of organic liquids. J. Am. Chem. Soc. 118, 11225–11236. 10.1021/ja9621760

[B22] Kaakoush N. O. Man S. M. Lamb S. Raftery M. J. Wilkins M. R. Kovach Z.. (2010). The secretome of *Campylobacter concisus*. FEBS J. 277, 1606–1617. 10.1111/j.1742-4658.2010.07587.x 20148967

[B23] Kaakoush N. O. Mitchell H. M. Man S. M. (2014). Role of emerging Campylobacter species in inflammatory bowel diseases. Inflamm. Bowel Dis. 20, 2189–2197. 10.1097/MIB.0000000000000074 24874462

[B24] Käll L. Krogh A. Sonnhammer E. L. L. (2004). A combined transmembrane topology and signal peptide prediction method. J. Mol. Biol. 338, 1027–1036. 10.1016/j.jmb.2004.03.016 15111065

[B25] Laskowski R. A. MacArthur M. W. Moss D. S. Thornton J. M. (1993). PROCHECK: a program to check the stereochemical quality of protein structures. J. Appl. Cryst. 26:283–291. 10.1107/S0021889892009944

[B26] Letchumanan V. Chan K. G. Khan T. M. Bukhari S. I. Mutalib N. S. A. Goh B. H.. (2017). Bile sensing: the activation of Vibrio parahaemolyticus virulence. Front. Microbiol. 8:728. 10.3389/fmicb.2017.00728 28484445 PMC5399080

[B27] Liu F. Lee H. Lan R. Zhang L. (2016). Zonula occludens toxins and their prophages in Campylobacter species. Gut Pathog. 8:43. 10.1186/s13099-016-0125-1 27651834 PMC5025632

[B28] Ma Y. J. Sun X. H. Xu X. Y. Zhao Y. Pan Y. J. Hwang C. A.. (2015). Investigation of reference genes in Vibrio parahaemolyticus for gene expression analysis using quantitative RT-PCR. PLoS ONE 10:e0144362. 10.1371/journal.pone.0144362 26659406 PMC4676679

[B29] Mahendran V. Liu F. Riordan S. M. Grimm M. C. Tanaka M. M. Zhang L. (2016). Examination of the effects of *Campylobacter concisus* zonula occludens toxin on intestinal epithelial cells and macrophages. Gut Pathog. 8:18. 10.1186/s13099-016-0101-9 27195022 PMC4870807

[B30] Mahoney J. C. Gerding M. J. Jones S. H. Whistler C. A. (2010). Comparison of the pathogenic potentials of environmental and clinical Vibrio parahaemolyticus strains indicates a role for temperature regulation in virulence. Appl. Environ. Microbiol. 76, 7459–7465. 10.1128/AEM.01450-10 20889774 PMC2976215

[B31] Marchler-Bauer A. Bo Y. Han L. He J. Lanczycki C. J. Lu S.. (2017). CDD/SPARCLE: functional classification of proteins via subfamily domain architectures. Nucl. Acids Res. 45, D200–D203. 10.1093/nar/gkw1129 27899674 PMC5210587

[B32] Martyna G. J. Tobias D. J. Klein M. L. (1994). Constant pressure molecular dynamics algorithms. J. Chem. Phys. 101, 4177–4189. 10.1063/1.467468

[B33] MINSAL (2017). Base de datos RANKIN-ETA DEIS, Ministerio de Salud. Avaliable online at: https://public.tableau.com/profile/deis4231#!/vizhome/BrotesdeEnfermedadesTransmitidasporAlimentoETA_Aos2011-2017/BrotesETAChile2011-2017 (accessed March 15, 2020).

[B34] Nettleton D. (2014). Chapter 6 - Selection of Variables and Factor Derivation in "Commercial data mining" Processing, in Analysis and Modeling for Predictive Analytics Projects. The Savvy Manager's Guides. (MA: Morgan Kaufmann Publishers Elsevier Inc), 79–104. 10.1016/B978-0-12-416602-8.00006-6

[B35] Nishibuchi M. Fasano A. Russell R. G. Kaper J. B. (1992). Enterotoxigenicity of Vibrio parahaemolyticus with and without genes encoding thermostable direct hemolysin. Infect. Immun. 60, 3539–3545. 10.1128/IAI.60.9.3539-3545.1992 1500161 PMC257358

[B36] Notredame C. Higgins D. G. Heringa J. (2000). T-coffee: a novel method for fast and accurate multiple sequence alignment. J. Mol. Biol. 302, 205–217. 10.1006/jmbi.2000.4042 10964570

[B37] Omasits U. Ahrens C. H. Müller S. Wollscheid B. (2014). Protter: interactive protein feature visualization and integration with experimental proteomic data. Bioinformatics 30, 884–886. 10.1093/bioinformatics/btt607 24162465

[B38] Pérez-Reytor D. García K. (2018). Galleria mellonella: a model of infection to discern novel mechanisms of pathogenesis of non-toxigenic Vibrio parahaemolyticus strains. Virulence 9, 22–24. 10.1080/21505594.2017.1388487 28981394 PMC5955188

[B39] Pfaffl M. W. Horgan G. W. Dempfle L. (2002). Relative expression software tool (REST(C)) for group-wise comparison and statistical analysis of relative expression results in real-time PCR. Nucl. Acids Res. 30:e36. 10.1093/nar/30.9.e36 11972351 PMC113859

[B40] Raghunath P. (2014). Roles of thermostable direct hemolysin (TDH) and TDH-related hemolysin (TRH) in Vibrio parahaemolyticus. Front. Microbiol. 5:805. 10.3389/fmicb.2014.00805 25657643 PMC4302984

[B41] Roy A. Kucukural A. Zhang Y. (2010). I-TASSER: a unified platform for automated protein structure and function prediction. Nat. Protoc. 5, 725–738. 10.1038/nprot.2010.5 20360767 PMC2849174

[B42] Saha S. Raghava G. P. S. (2007). BTXpred: prediction of bacterial toxins. In Silico Biol. 7, 405–412. Available online at: https://pubmed.ncbi.nlm.nih.gov/18391233/18391233

[B43] Schmidt E. Kelly S. M. van der Walle C. F. (2007). Tight junction modulation and biochemical characterisation of the zonula occludens toxin C-and N-termini. FEBS Lett. 581, 2974–2980. 10.1016/j.febslet.2007.05.051 17553496

[B44] Shinoda S. (2011). Sixty years from the discovery of Vibrio parahaemolyticus and some recollections. Biocontrol Sci. 16, 129–137. 10.4265/bio.16.129 22190435

[B45] Steele-Mortimer O. Knodler L. A. Brett Finlay B. (2000). Poisons, ruffles and rockets: bacterial pathogens and the host cell cytoskeleton. Traffic 1, 107–118. 10.1034/j.1600-0854.2000.010203.x 11208091

[B46] Tanabe T. Miyamoto K. Tsujibo H. Yamamoto S. Funahashi T. (2015). The small RNA Spot 42 regulates the expression of the type III secretion system 1 (T3SS1) chaperone protein VP1682 in Vibrio parahaemolyticus. FEMS Microbiol. Lett. 362:fnv173. 10.1093/femsle/fnv173 26394644

[B47] Uzzau S. Cappuccinelli P. Fasano A. (1999). Expression of Vibrio cholerae zonula occludens toxin and analysis of its subcellular localization. Microb. Pathog. 27, 377–385. 10.1006/mpat.1999.0312 10588910

[B48] Uzzau S. Lu R. Wang W. Fiore C. Fasano A. (2001). Purification and preliminary characterization of the zonula occludens toxin receptor from human (CaCo2) and murine (IEC6) intestinal cell lines. FEMS Microbiol. Lett. 194, 1–5. 10.1111/j.1574-6968.2001.tb09437.x 11150657

[B49] Vanuytsel T. Vermeire S. Cleynen I. (2013). The role of Haptoglobin and its related protein, Zonulin, in inflammatory bowel disease. Tissue Barriers 1:e27321. 10.4161/tisb.27321 24868498 PMC3943850

[B50] Wagley S. Borne R. Harrison J. Baker-Austin C. Ottaviani D. Leoni F.. (2018). Galleria mellonella as an infection model to investigate virulence of Vibrio parahaemolyticus. Virulence 9, 197–207. 10.1080/21505594.2017.1384895 28960137 PMC5801645

[B51] Waterhouse A. M. Procter J. B. Martin D. M. A. Clamp M. Barton G. J. (2009). Jalview version 2–a multiple sequence alignment editor and analysis workbench. Bioinformatics 25, 1189–1191. 10.1093/bioinformatics/btp033 19151095 PMC2672624

[B52] Wiederstein M. Sippl M. J. (2007). ProSA-web: interactive web service for the recognition of errors in three-dimensional structures of proteins. Nucl. Acids Res. 35, W407–W410. 10.1093/nar/gkm290 17517781 PMC1933241

[B53] Yang J. Zhang Y. (2015). I-TASSER server: new development for protein structure and function predictions. Nucl. Acids Res. 43, W174–W181. 10.1093/nar/gkv342 25883148 PMC4489253

[B54] Yu Y. Yang H. Li J. Zhang P. Wu B. Zhu B.. (2012). Putative type VI secretion systems of Vibrio parahaemolyticus contribute to adhesion to cultured cell monolayers. Arch. Microbiol. 194, 827–835. 10.1007/s00203-012-0816-z 22535222

[B55] Zhang L. Orth K. (2013). Virulence determinants for Vibrio parahaemolyticus infection. Curr. Opin. Microbiol. 16, 70–77. 10.1016/j.mib.2013.02.002 23433802

[B56] Zhang L. Lee H. Grimm M. C. Riordan S. M. Day A. S. Lemberg D. A. (2014). *Campylobacter concisus* and inflammatory bowel disease. World J. Gastroenterol. 20, 1259–1267. 10.3748/wjg.v20.i5.1259 24574800 PMC3921508

[B57] Zhang Y. (2008). I-TASSER server for protein 3D structure prediction. BMC Bioinformatics 9:40. 10.1186/1471-2105-9-40 18215316 PMC2245901

