# Supplementary material for: Analysis of the Zonula occludens Toxin Found in the Genome of the Chilean Non-toxigenic Vibrio parahaemolyticus Strain PMC53.7
Source: Front Cell Infect Microbiol. 2020 Sep 24;10:482. doi: 10.3389/fcimb.2020.00482 (PMC7541967; doi:10.3389/fcimb.2020.00482)
Supplement: Supplementary file 1 [file Data_Sheet_1.docx]

**Supplementary Figures**

| M.W  Ara^-^  C-term Ara^-^ Zot-PMC53.7  (kDa)  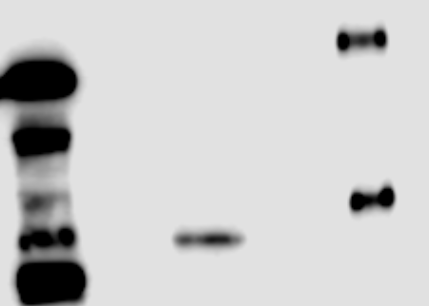  22 kDa  27 kDa  57 kDa |
| --- |

**Supplementary Figure 1.** Recombinant Zot (Zot-PMC53.7) and C-terminal (C-term) fragment expression detected by western blot with mouse anti-His antibodies. Recombinant proteins were not detected in the empty vector controls**.** 6xHis-Zot fusion proteins were expressed in *E. coli* BL21(DE3)/pBAD33.1 Ara- means non-induction with arabinose.

**
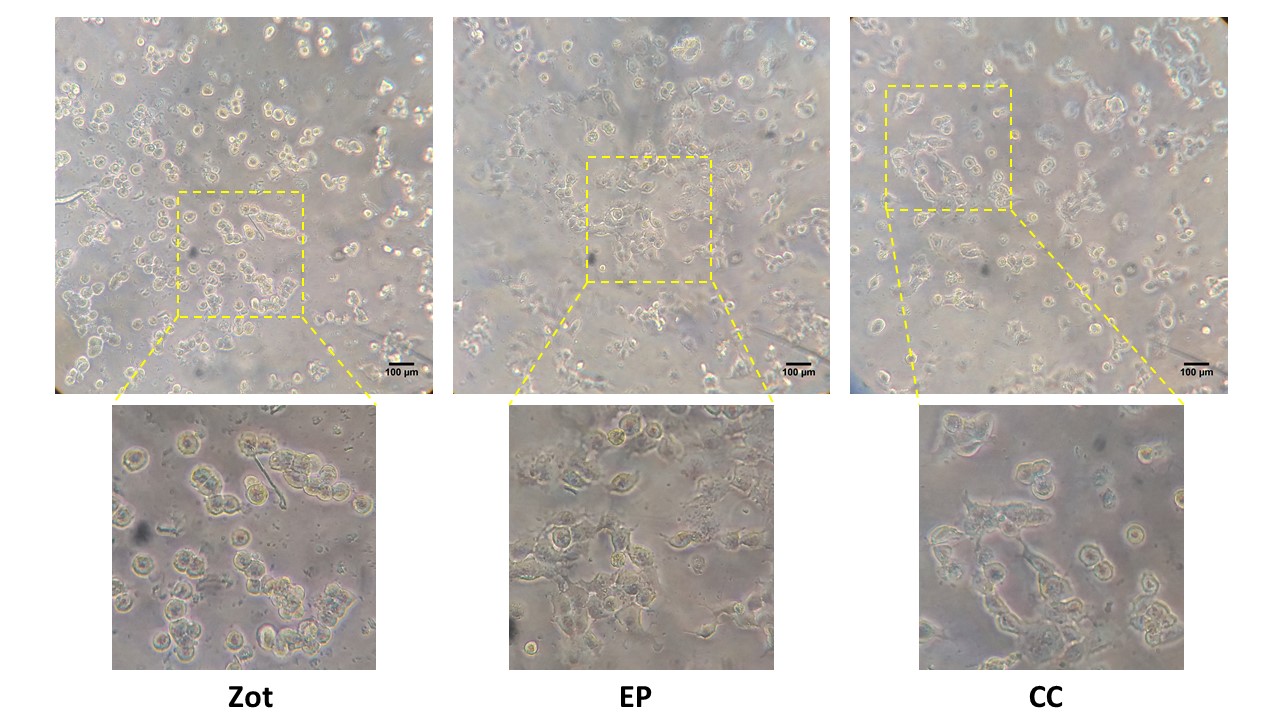
**

**Supplementary Figure 2.** Impairment of cellular attachment to the plate surface was observed at 4 h post-treatment of Caco-2 cells with PMC53.7-Zot but was not observed with *E. coli* proteins (EP) control. Cellular control (CC) shows the normal cellular attachment.


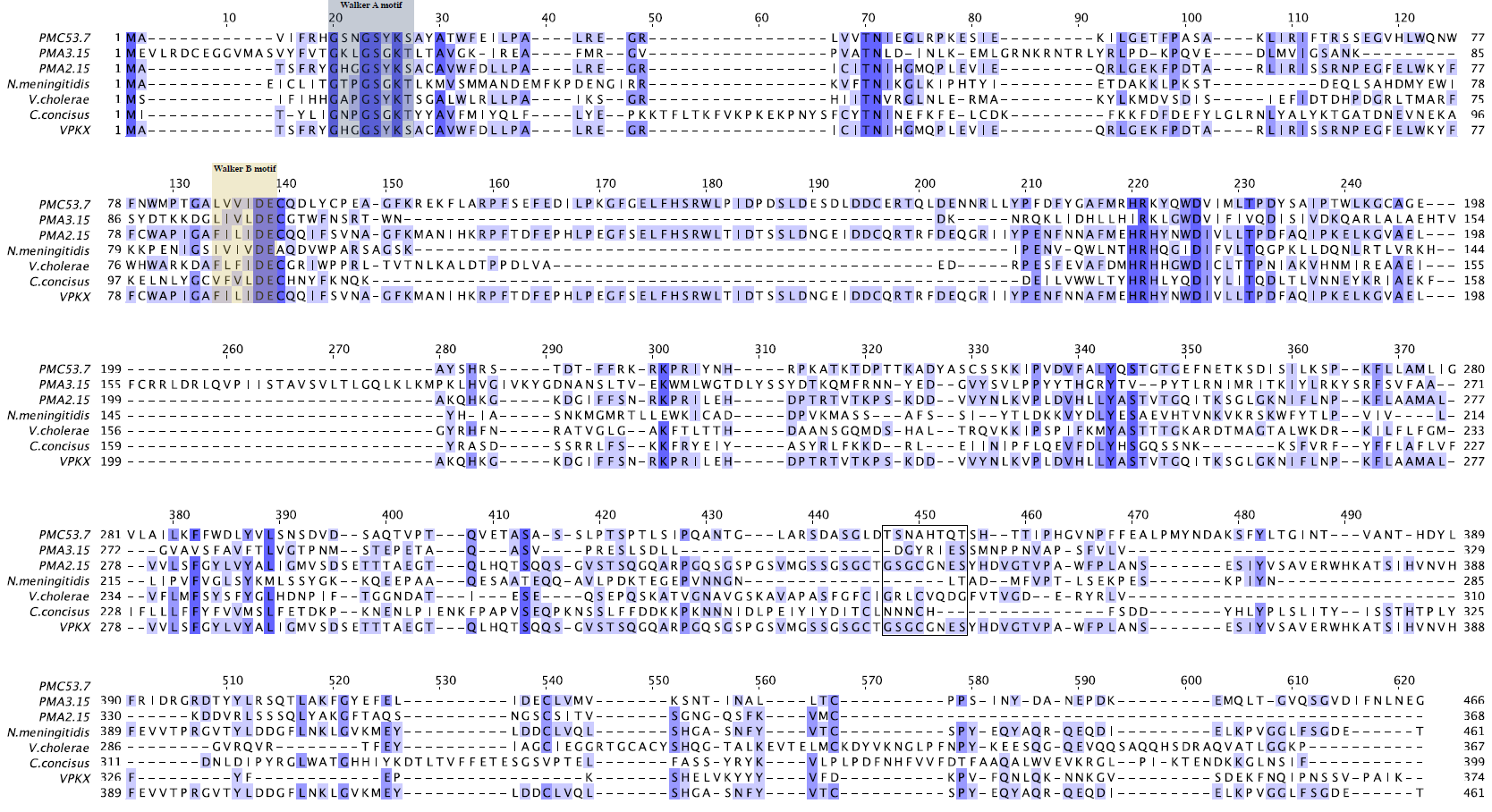


**Supplementary Figure 3:** Multiple Sequence Alignment comparing Zot of different species of human pathogens.


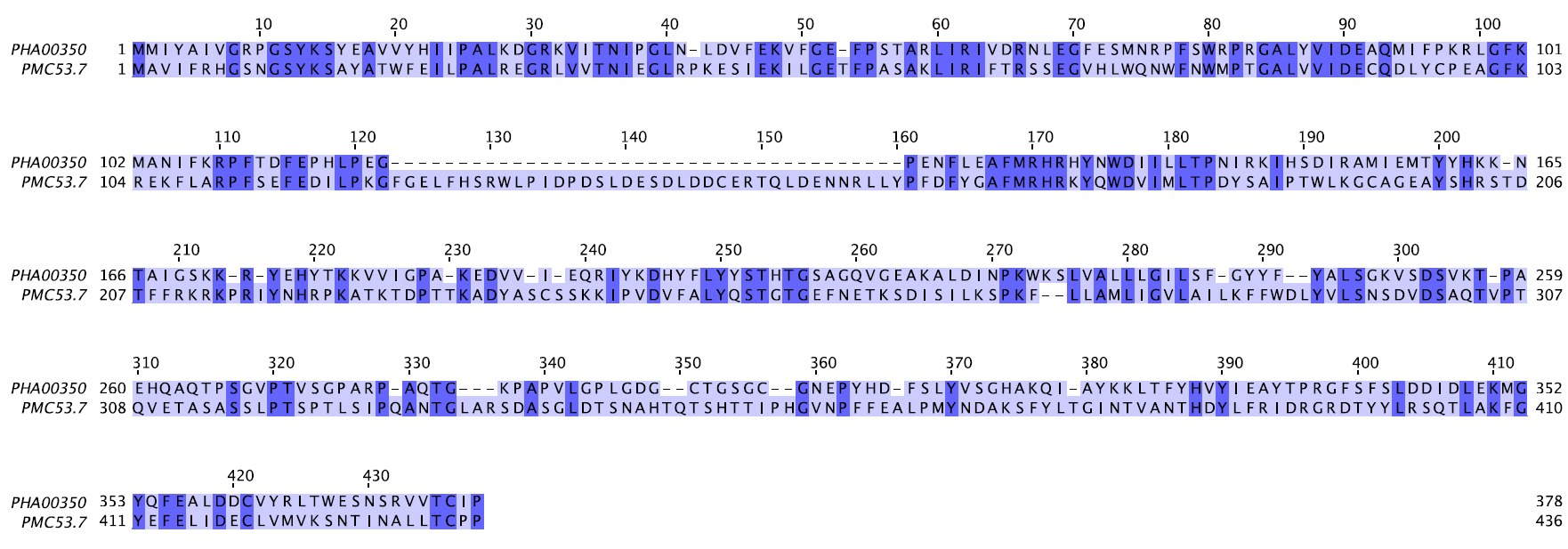


**Supplementary Figure 4:** Alignment of the Zot sequence of *V. parahaemolyticus* PMC53.7 against protein sequence of PHA00350, member of the P-loop NTPase Superfamily.

**Supplementary Figure 5.** **PMC53.7 bioinformatics predictions. A**. Protein topology showing N-terminal cytosolic domain, C-terminal extracellular domain, and transmembrane domain. Walker motifs A and B are displayed as blue and yellow residues, respectively. **B.** Phosphorylation sites predicted in the PMC53.7 protein, residues whose values are higher than the threshold represent the potential phosphorylation sites. **C.** Secondary structure predicted in the PMC53.7-Zot, the 𝛼-helices and β-sheets are represented in red and green, respectively **D**. Prediction of cytoplasmic, extracellular and transmembrane domains calculated by Phobius server. **E**. Accessible surface area, values range from 0 (buried residue) to 8 (highly exposed residue). **F**. Regional hydropathy, the black line represents the hydrophobicity of each residue obtained by using the Kyte-Doolittle algorithm. The blue line represents a normalized consensus hydrophobicity calculated by using the Eisenberg algorithm. **G**. Normalized β-Factor, indicating the extent of the inherent thermal mobility of each residue.


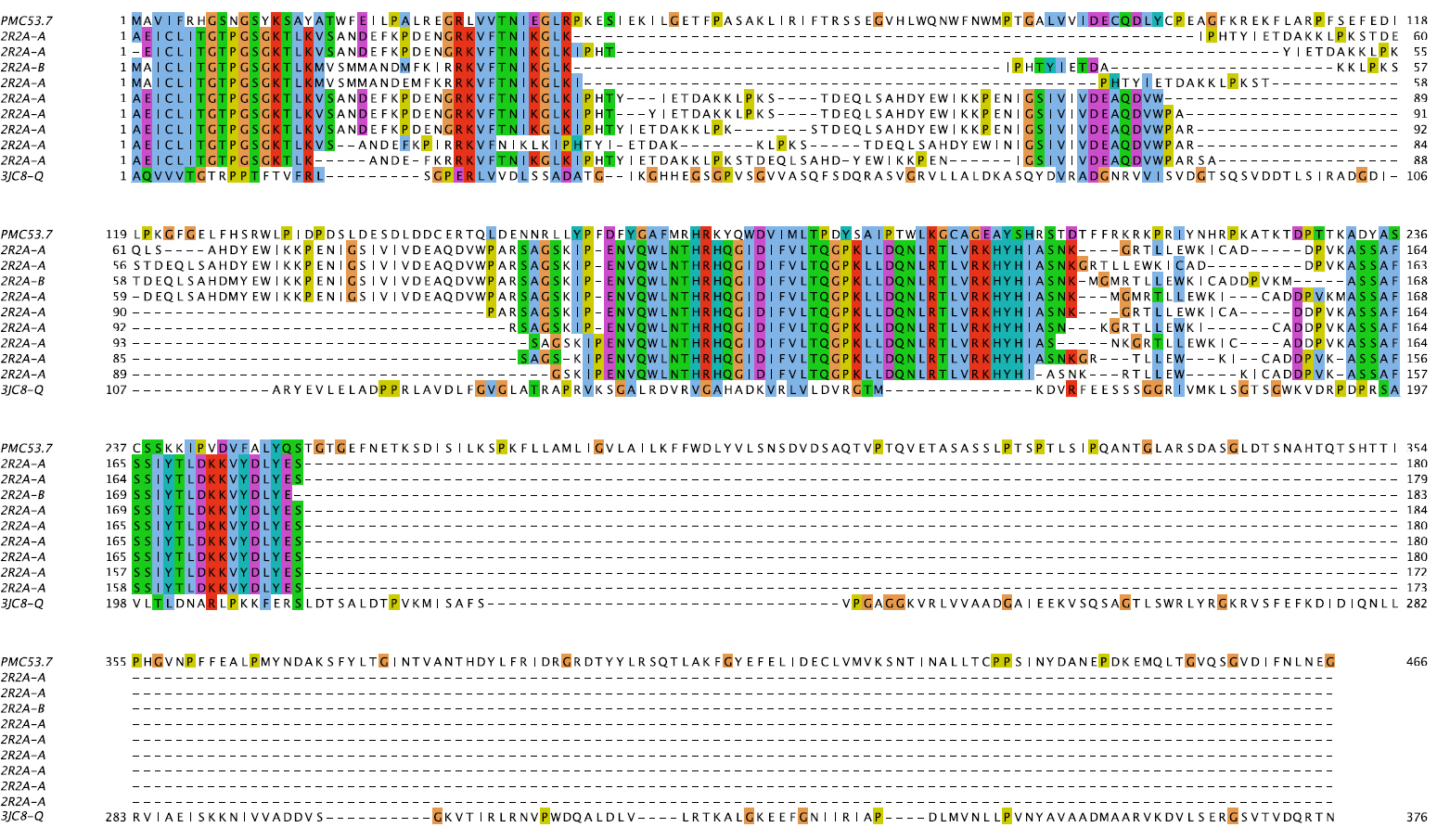


**Supplementary Figure 6:** Multiple sequence alignment generated by I-Tasser using two suitable templates (PDB codes: 2R2A and 3JC8) to generate multiple PMC53.7 models.


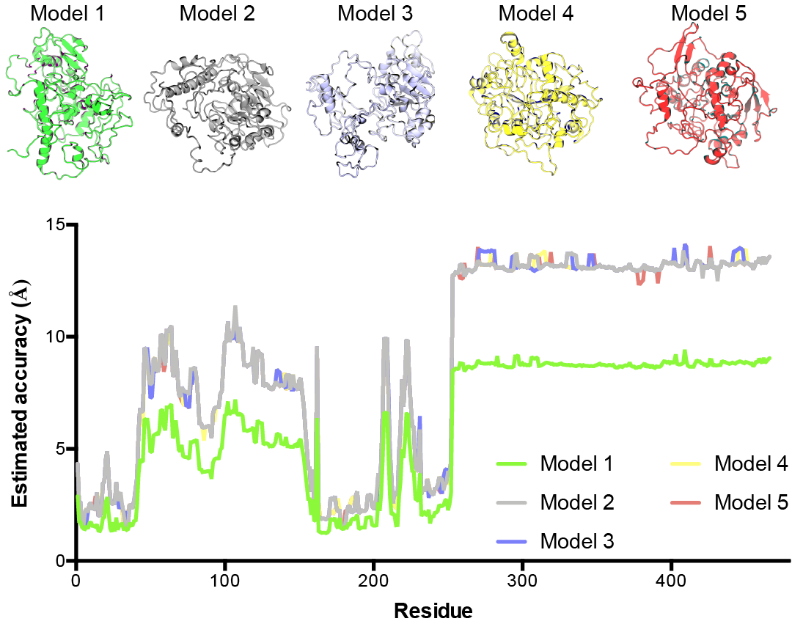


**Supplementary Figure 7**. **PMC53.7-Zot models generated.** The top-5 models generated by I-TASSER are displayed (top-panel), The estimated local accuracy between residue position in the models and native structure, in Angstroms (A°) is displayed (bottom-panel).

**Supplementary Figure 8.** **Analysis of the 250ns-MDs of PMC53.7-Zot.** **A**. Time dependence of the RMSD for backbone atoms of PMC53.7 model. **B**. Time dependence of the RMSD for backbone atoms of PMC53.7 domains. **C**. Root Mean Square Fluctuation (RMSF) characterizing the internal fluctuation of PMC53.7 residues during the simulation time.

**Supplementary Figure 9:** PMC53.7-Zot model exhibiting high percentage of the residues in the most favored regions of the Ramachandran plots before and after refinement.


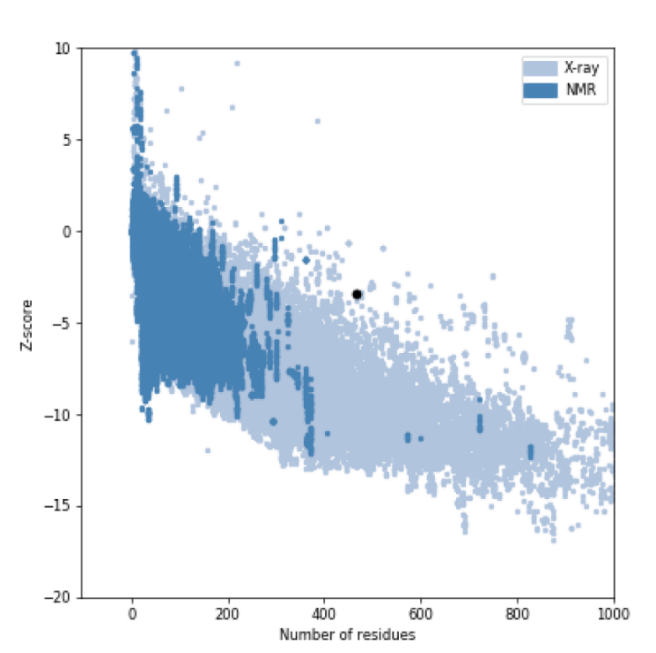


**Supplementary Figure 10:** Z-score of the PMC53.7-Zot stabilized model after 250 ns of MDs.

**Supplementary Figure 11.** Analysis of the PMC53.7-Zot protein secondary structure elements (SSE). **A.** %SSE in PMC53.7 residues. **B.** SSE assignment for each residue over the 250ns-MDs. **C.** 3D representation of the equilibrated PMC53.7 model after 250ns (last frame). The 𝛼-helices and β- strands are displayed in orange and blue, respectively.
